# Long Acting and Highly Effective Pre-Exposure Prophylaxis (Prep) Using Injectable Lenacapavir (LEN) for HIV Infection

**DOI:** 10.34172/apb.46524

**Published:** 2026-05-10

**Authors:** Mariyam Niusha Naseer, Fathimath Ina Shareef, Ismail Junaih Shareef, Kannan Subbaram, Razana Faiz, Sheeza Ali, Queen Alice Arul

**Affiliations:** ^1^School of Medicine, the Maldives National University, Male’, Maldives; ^2^Indira Gandhi Memorial Hospital, Male’, Maldives; ^3^All India Institute of Medical Sciences, Kalyani, India

## To Editor,

 Human immunodeficiency virus (HIV) is one of the major public health concerns worldwide. In 2024, 1.3 million new cases of HIV were reported, bringing the global burden to 40.8 million. Approximately 630,000 individuals died from HIV-related causes in the same year.^1^ Although effective antiretroviral therapy has transformed HIV into a chronic, treatable infection, the path to ending the public health threat posed by HIV is through prevention.^2^ One highly effective prevention option for populations at substantial risk is pre-exposure prophylaxis (PrEP), which has been shown to significantly reduce the incidence of HIV infections.^3,4^

 Previously, PrEP options were limited to tenofovir based oral PrEP, dapivirine (DVR) and long acting bimonthly injectable cabotegravir (CAB-LA).^5^ Recently, the United States Food and Drug Administration (FDA) has approved the use of lenacapavir (LEN) for HIV PrEP within the US.^6^ At the 13th International AIDS Society Conference (IAS 2025) on HIV science, LEN was officially included in WHO Guidelines as a recommended PrEP option.^7^

 LEN is a first-in-class, long-acting, ultra-potent viral capsid inhibitor. It functions by directly binding to the interface between HIV-1 capsid protein subunits, thereby selectively disrupting capsid function (Figure 1). The resulting virions have defective capsids and reduced infectivity.^8,9^

**Figure 1:**
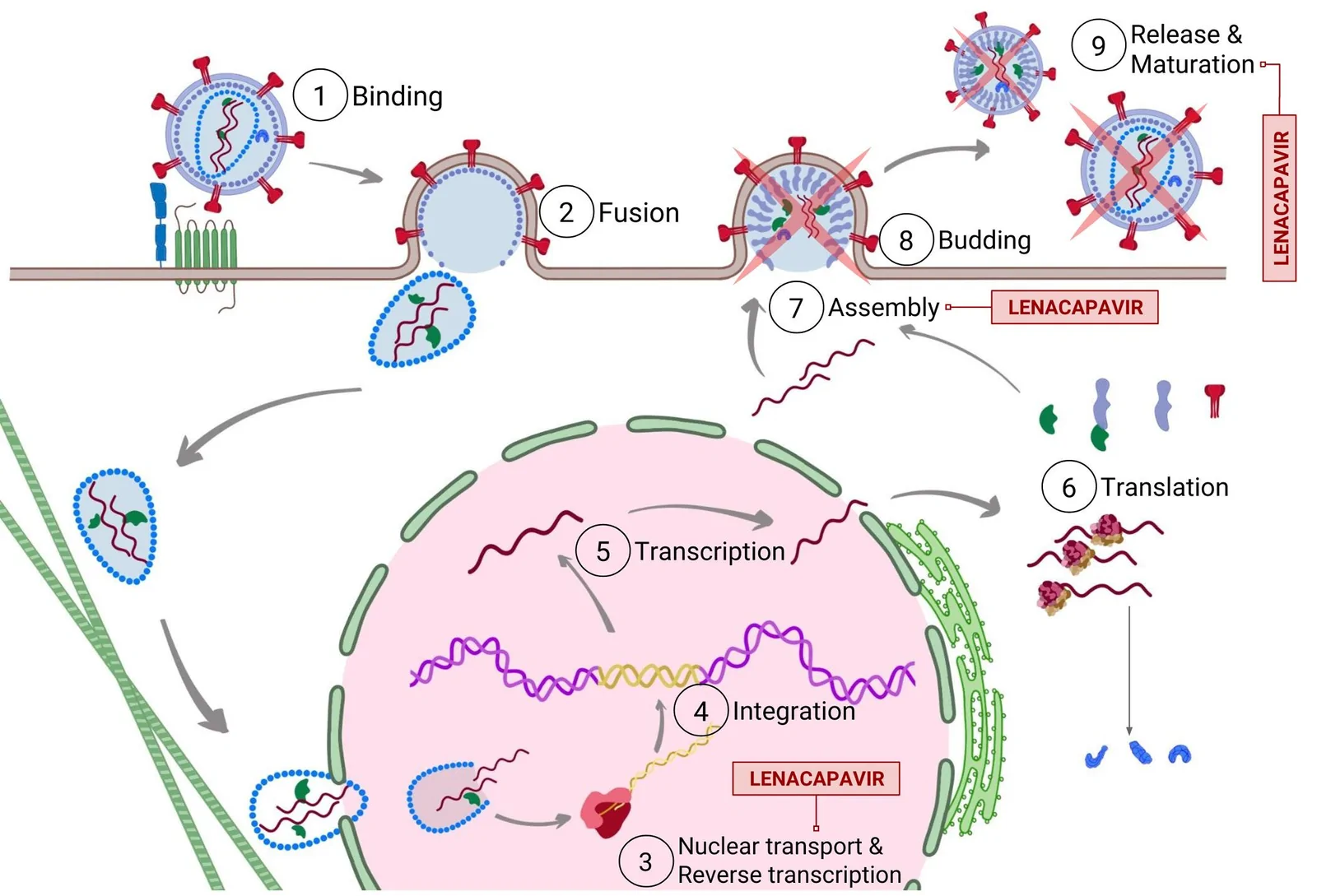
Mechanism of action of Lenacapavir (LEN) on HIV replication (image adapted from Van Heuvel Y et al. 2022^8^)

 The efficacy of LEN as PrEP among cisgender women in South Africa and Uganda was evaluated in the PURPOSE 1 trial. This was a double-blind, randomized, controlled trial involving 5338 participants. The incidence of HIV infection was compared with the estimated background incidence in the screened population. There were 0 infections among the 2134 participants in the Lenacapavir group (0 per 100 person-years) compared to a background HIV incidence of 2.41 per 100 person-years. This represented 100% efficacy (95% CI: 90.7–100.0) relative to background incidence.^10^

 The PURPOSE 2 trial evaluated the efficacy of semiannual LEN among 3,250 cisgender men, transgender and non-binary individuals. Among 3265 participants, HIV infections occurred in 2 individuals (0.10 per 100 person-years; 95% confidence interval [CI], 0.01 to 0.37) and in 9 participants in the F/TDF group (0.93 per 100 person-years; 95% CI, 0.43 to 1.77). Background HIV incidence in the screened population (4634 participants) was 2.37 per 100 person-years (95% CI, 1.65 to 3.42). While these results are promising, PURPOSE 3, 4 and 5 are currently underway to evaluate the efficacy of Lenacapavir as a HIV PrEP among broader demographics.^11,12^

 Despite its efficacy, use of Lenacapavir as a PrEP requires rigorous safeguards to be in place. For instance, HIV testing is required to exclude an active infection prior to each injection. If PrEP is initiated during the seroconversion window, there is high risk of developing capsid-inhibitor resistance. Moreover, to prevent resistance, the “pharmacokinetic tail” should be managed. This is the period when after stopping a drug, it remains detectable but sub-therapeutic. During this period, switching patients to a daily oral PrEP for tail coverage can help prevent resistance. Furthermore, patients should be counseled that this does not protect against other sexually transmitted infections, therefore safe sex precautions must be taken while on Lenacapavir as well. Some injection site reactions such as nodules have been reported. To prevent this, patients must be educated on correct injection techniques.^13^ Clinicians must also keep in mind the drug-drug interactions. Drugs which are strong or moderate CYP3A inducers significantly decrease plasma concentrations of Lenacapavir.^14^

## Conclusion

 Semiannual dosing regimen of Lenacapavir is a convenient new regimen that may improve adherence to PrEP regimens. Its usage requires continued monitoring and pharmacovigilance. However, it is a promising tool to reduce the public health threat posed by AIDS.

## Competing Interests

 Authors declare there are no competing interests.

## Ethical Approval

 Not applicable.
